# Symphysis Pubis Osteomyelitis with Bilateral Adductor Muscles Abscess

**DOI:** 10.1155/2014/982171

**Published:** 2014-12-14

**Authors:** Saad M. Alqahtani, Fan Jiang, Bardia Barimani, Marie Gdalevitch

**Affiliations:** ^1^Division of Orthopaedic Surgery, Shriners Hospital for Children, Montreal Children Hospital, McGill University, 1529 Cedar Avenue, Montreal, QC, Canada H3G 1A6; ^2^Department of Orthopedic Surgery, University of Dammam, Dammam 31451, Saudi Arabia; ^3^Imperial College London, London SW7 2AZ, UK

## Abstract

Osteomyelitis of the pubis symphysis is a rare condition. There have been various reports in the literature of inflammation and osteomyelitis as well as septic arthritis of pubic symphysis. However, due to the fact that these conditions are rare and that the usual presenting symptoms are very nonspecific, osteomyelitis of the pubic symphysis is often misdiagnosed, thus delaying definitive treatment. We present a case that to our knowledge is the first case in literature of osteomyelitis of the pubic symphysis in a 17-year-old boy with juvenile idiopathic arthritis (JIA), which was initially misdiagnosed and progressed to bilateral adductor abscesses. A high suspicion of such condition should be considered in a JIA patient who presents with symphysis or thigh pain.

## 1. Introduction

Osteomyelitis of the pubis symphysis is a rare condition that occurs in less than 1% of cases of osteomyelitis. There have been various reports in the literature of inflammation and osteomyelitis as well as septic arthritis of pubic symphysis in patients during pregnancy, after delivery, and after surgical procedure and other invasive procedures and intravenous drug users [1–7]. There have also been reports of the condition in young, healthy patients and athletes [8–11]. However, due to the fact that these conditions are rare and that the usual presenting symptoms are very nonspecific, osteomyelitis of the pubic symphysis is often misdiagnosed, thus delaying definitive treatment. We present a case that to our knowledge is the first case in literature of osteomyelitis of the pubic symphysis in a 17-year-old boy known for juvenile idiopathic arthritis (JIA), which was initially misdiagnosed and progressed to bilateral adductor abscesses.

## 2. Case Presentation

Our patient is known for a 2-year history of JIA treated with regular methotrexate and diclofenac. He experienced groin pain for 3 weeks, which was exacerbated with walking. He presented to the emergency department with severe groin pain following an intense exercise class. He was initially diagnosed with postexercise myositis. The pain however was persistent for the next 2 weeks, associated with night chills and fevers. During this time period he also experienced a sore throat for a week, which resolved without treatment.

**Figure 1:**
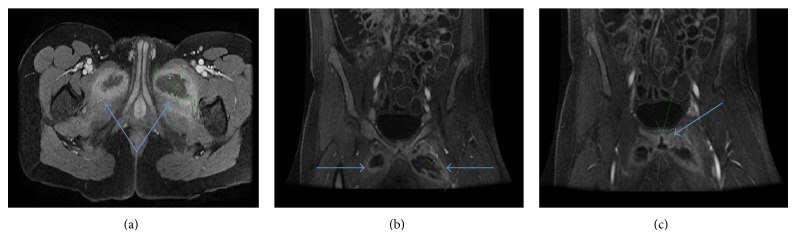
Preoperative MRI showing ((a), (b)) large fluid collection within the medial compartment of the thigh bilaterally, extending along the adductor brevis and adductor longus muscles. (c) The abscess on the left side extending more superiorly to the midline with fluid in the symphysis pubis joint suggestive of an effusion and symphysis.

Patient presented to his rheumatologist who thought his symptoms were likely due to myositis or possibly a flare-up of his JIA and he was given steroids treatment. During this period, he had an MRI enterography organized by his rheumatologist for a work-up of inflammatory bowel disease, which was suspected due to previous bowel problems. Aside from local abnormal wall thickening of the terminal ileum, the MRI also showed pubic bone osteomyelitis, large bilateral abscesses in the medial compartment of the thighs measuring 1.3 × 3.7 × 8.7 cm on left and 5.4 × 3.2 × 5.3 on right. Upon reviewing the imaging, patient's rheumatologist contacted him and requested that he go to the emergency department (Figure 1).

On his second visit to emergency department, he looked well and was afebrile. His vital signs were stable aside from slight tachycardia. Aside from mild lymphadenopathy in his neck, the rest of systemic exam was within normal limits. He was able to weight bear with moderate amounts of pain in the left hip; his range of motion was full bilaterally in the lower extremity with moderate pain when ranging his left hip. Laboratory data revealed peripheral white blood cell count of 19.1, absolute neutrophil count of 14.6, C-reactive protein (CRP) of 232, and erythrocyte sedimentation rate (ESR) of 57. Blood cultures from the emergency department were pending.

A single dose of intravenous Tazocin 400 mg was given in the emergency. Orthopedics and the infectious disease team were consulted. Due to the development of bilateral adductor abscess and questionable joint involvement on MRI, we proceeded with an irrigation and debridement of the bilateral abscess; we did an intraoperative aspiration of the hip joint in question which did not appear to be infected and thus we did not perform arthrotomy. Bilateral medial longitudinal incisions were made in the adductor crease and the large collection of pus was found on both sides lying between adductor longus and pectineus. Intraoperative cultures were sent to the laboratory and was positive for group A streptococci. Antibiotics were continued postoperatively and were switched from Tazocin to Cefazolin 3 g IV every 8 hours by the infectious disease team.

**Figure 2:**
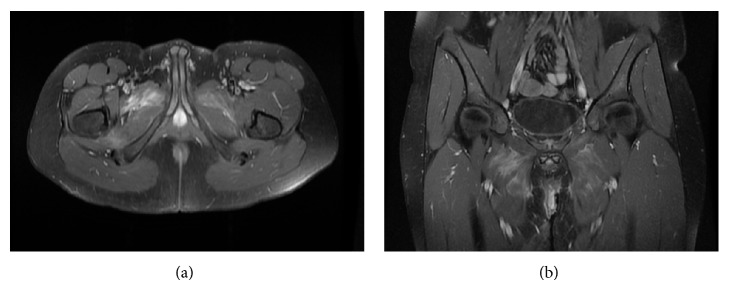
Postoperative MRI (a) axial, (b) coronal showing no collection and just residual edema and myositis 6 months after drainage.

After surgery, patient improved significantly and, on postoperative day one, the patient was already afebrile, no longer having bilateral groin pain, and was able to ambulate. He was discharged home with prescription of 6-week course of intravenous Ceftriaxone 400 mg once a day. His methotrexate was held until completion of his course of 6 weeks of antibiotics treatment. On follow-up visits, we observed that he made a quick clinical and radiological recovery (Figure 2) and has been well since.

## 3. Discussion

Ross and Hu, 2003, reviewed 100 cases of pubic symphysis septic arthritis [12]. They demonstrated that this condition is not specific to any age group and can range from 7 to 86 years of age. Most common presenting signs and symptoms include fever, pubic tenderness, antalgic gait, and pain with active/passive range of motion of hip. They identified several predisposing factors including urological, gynecological procedures, pregnancy, athletes, malignancy, and intravenous drug use, as well as other invasive procedures.

Interestingly, our patient was a 17-year-old boy known for JIA and presented with septic arthritis of the pubic symphysis and bilateral adductor abscesses. Our case further demonstrated the vague nonspecific symptoms of infection of pubic symphysis which can be misleading on initial presentation. Symptoms of pubic, groin, and hip tenderness and antalgic gait as well as elevated inflammatory markers can mimic a condition known as osteitis pubis, which is an inflammatory condition of the pubic symphysis often seen in athletes, pregnancy, and trauma as well as posturological and postgynecological operations. The differentiating factor between osteitis pubis and osteomyelitis of the pubis symphysis is often a negative culture on biopsy [13]. The most common pathogen causing infections of pubis symphysis was found to be* Staphylococcus aureus*; however,* Pseudomonas aeruginosa*,* Escherichia coli*,* Enterococcus *sp.,* Mycobacterium tuberculosis*,* Salmonella *sp., and* Streptococcus *sp., as well as others have also been reported in literature [12, 13].

It is difficult clinically to identify infection of the pubis symphysis, as the symptoms are very nonspecific and do not present like a typical septic arthritis of a joint such as knee or shoulder. However, we recommend having a high level of suspicion for patients presenting with pubic, groin, and hip pain, especially if patient is immunosuppressed. X-ray of pelvis is insensitive in terms of findings for osteomyelitis of the pubic symphysis [12], when suspicious imagining modalities such as computed tomography as well as magnetic resonance imagining should be employed. To our knowledge, no randomized control trial exists for comparing the different treatment modalities. It has been reported in literature that patients with osteomyelitis of pubis symphysis can be successfully treated with intravenous and/or oral antibiotics; however, with the development of any abscess, surgical intervention is usually required [14]. In our patient, antibiotic was started in the emergency department prior to orthopedic consultation. We recommend starting antibiotic during surgery immediately after culture is obtained for microbiology. Thus, it is important that these infections are identified early before the development of abscesses as surgical or radiological intervention might then be warranted.

In summary, osteomyelitis of pubic symphysis is a rare condition that is often missed due to its nonspecific presentation. It can be effectively treated with antibiotics at the early stage; however, when abscess develops, a surgical intervention is usually warranted. Thus, we recommend keeping a high level of suspicion in patients presenting with symptoms of pubic, groin, and hip pain after ruling out any traumatic fractures or other causes. Lastly, to our knowledge, there is no consensus as to the duration of antibiotic treatment for osteomyelitis of pubic symphysis. We believe that it would be an important study to look into in the near future.
